# Complete Genome Sequence of *Rhodococcus* sp. Strain M8, a Platform Strain for Acrylic Monomer Production

**DOI:** 10.1128/MRA.01314-20

**Published:** 2021-03-11

**Authors:** Andrey D. Novikov, Konstantin V. Lavrov, Artem S. Kasianov, Maxim A. Topchiy, Tatyana V. Gerasimova, Alexander S. Yanenko

**Affiliations:** a NRC Kurchatov Institute-GosNIIgenetika, Kurchatov Genomic Center, Moscow, Russia; b NRC Kurchatov Institute, Moscow, Russia; c Vavilov Institute of General Genetics, Moscow, Russia; d A. V. Topchiev Institute of Petrochemical Synthesis, Russian Academy of Sciences, Moscow, Russia; University of Southern California

## Abstract

We report a 6.27-Mbp complete genome of *Rhodococcus* sp. strain M8, an originally discovered strain that is now under investigation for production of acrylic monomers. The genome consists of a 6.1-Mbp circular chromosome and a 173.2-kbp plasmid.

## ANNOUNCEMENT

Here, we present a complete sequence of the Rhodococcus rhodochrous strain M8 genome, which was obtained after Nanopore *de novo* sequencing and joining of this sequence with the previously obtained draft genome sequence of M8 (1). The effort to close this genome was undertaken because the strain is actually a platform for the development of biocatalysts for acrylic monomer production (several biocatalysts were derived from it earlier; see references 2–4 and also see the information about biocatalytic production of acrylic monomers [5, 6]). Additionally, the strain is one of the model strains for basic research on the genetics of rhodococci (1, 7–9). Further basic and applied research based on the strain requires precise genome information. Together with that, rhodococcal genomes are significantly variable in whole-genome size and the presence of genes for secondary metabolism. The quantity of complete rhodococcal genomes is too low now (five genomes [10]); therefore, this new genome is a valuable contribution to the knowledge base in this field.

The genomic DNA was extracted from a culture grown in Luria broth and purified using the Puregene Yeast/Bact. kit B (Qiagen), and PCR-free libraries were made with a ligation sequencing kit (SQK-LSK109), according to the manufacturer’s protocol for native barcoding of genomic DNA. The libraries were sequenced using an Oxford Nanopore Technologies (ONT) platform with a MinION R9 flow cell by Genotek (Moscow, Russia), and FAST5 files were generated. Base calling was performed on a local system using ONT Guppy software v3.4.1 with GPU support (https://github.com/gnetsanet/ONT-GUPPY), with default parameters. The resulting FASTQ file was used for adapter trimming with Porechop v0.2.4 (https://github.com/rrwick/Porechop) with default parameters. After that, 133,682 reads were obtained, with an *N*_50_ value of 12.3 kbp. Low-quality nucleotides and short reads were removed using Cutadapt v2.7 (11) with the parameters -q 20 -m 1000. Thus, 93,190 high-quality reads were obtained. These reads were corrected using Canu v1.7 (12) with the parameters -correct -nanopore genomeSize = 6000k. The corrected reads obtained had an average length of 8,382 nucleotides (nt) and a maximal length of 77,355 nt. These reads were used for *de novo* genome assembly using Flye v2.7 (13) with default parameters, resulting in two contigs of 6.1 and 0.17 Mbp. Finally, Illumina reads obtained previously (1) (also see the link to the SRA record below) were mapped to the Nanopore assembly using BWA MEM v0.7.15 (14) with default parameters. The BAM file obtained was used for genome polishing with Pilon v1.23 (15) with default parameters. After these procedures, the two contigs mentioned above remained separate DNAs. These 6.1- and 0.17-Mbp contigs were checked for circularity using Circlator (16), with default parameters. As a result, the 6.1-Mbp contig was shown to be a circular 6,106,346-nt chromosome, while the 0.17-Mbp contig remained not closed to the circle. The latter was also checked manually for the absence of overlapping ends. We suggest that the 0.17-Mbp contig could be a linear 173,200-nt plasmid. The average coverage for both contigs was estimated as 236-fold, and the complete genome was not rotated to any certain base. Finally, we checked that 93% of all Nanopore reads and 95% of all Illumina reads were mapped on the completed genome. Gene predictions and annotations were performed using the NCBI Prokaryotic Genome Annotation Pipeline (PGAP) (17), and 5,708 genes, including 5,639 coding sequences, were predicted.

### Data availability.

This genome sequence has been deposited in NCBI GenBank with accession number GCA_015654185. Raw sequencing reads used in the work are available under SRX9609528 (Nanopore) and SRR13296353 (Illumina). The version of the complete genome described in this paper is the first version, GCA_015654185.1.

## References

[1] Novikov AD, Lavrov KV, Kasianov AS, Gerasimova TV, Yanenko AS. 2018. Draft genome sequence of *Rhodococcus* sp. strain M8, which can degrade a broad range of nitriles. Genome Announc 6:e01526-17. doi:10.1128/genomeA.01526-17. 29439044 PMC5805882

[2] Leonova TE, Astaurova OB, Ryabchenko LE, Yanenko AS. 2000. Nitrile hydratase of *Rhodococcus*. Appl Biochem Biotechnol 88:231–241. doi:10.1385/ABAB:88:1-3:231.

[3] Lavrov KV, Larikova GA, Yanenko AS. 2013. Novel biocatalytic process of N-substituted acrylamide synthesis. Appl Biochem Microbiol 49:702–705. doi:10.1134/S0003683813080048.

[4] Lavrov KV, Grechishnikova EG, Shemyakina AO, Novikov AD, Kalinina TI, Epremyan AS, Glinskii SA, Minasyan RA, Voronin SP, Yanenko AS. 2019. Optimization of the expression of nitrilase from *Alcaligenes denitrificans* in *Rhodococcus rhodochrous* to improve the efficiency of biocatalytic synthesis of ammonium acrylate. Appl Biochem Microbiol 55:861–869. doi:10.1134/S0003683819090035.

[5] Yanenko A, Osswald S. 2012. Hydrolysis of nitriles to amides, p 531–544. *In* Drauz K, Gröger H, May O (ed), Enzyme catalysis in organic synthesis. Wiley-VCH, Weinheim, Germany. doi:10.1002/9783527639861.ch13.

[6] Oßwald S, Yanenko A. 2012. Hydrolysis of nitriles to carboxylic acids, p 545–559. *In* Drauz K, Gröger H, May O (ed), Enzyme catalysis in organic synthesis. Wiley-VCH, Weinheim, Germany. doi:10.1002/9783527639861.ch14.

[7] Lavrov KV, Shemyakina AO, Grechishnikova EG, Novikov AD, Derbikov DD, Kalinina TI, Yanenko AS. 2018. New *cblA* gene participates in regulation of cobalt-dependent transcription of nitrile hydratase genes in *Rhodococcus rhodochrous*. Res Microbiol 169:227–236. doi:10.1016/j.resmic.2018.03.006. 29800680

[8] Lavrov KV, Shemyakina AO, Grechishnikova EG, Novikov AD, Kalinina TI, Yanenko AS. 2019. In vivo metal selectivity of metal-dependent biosynthesis of cobalt-type nitrile hydratase in *Rhodococcus* bacteria: a new look at the nitrile hydratase maturation mechanism? Metallomics 11:1162–1171. doi:10.1039/c8mt00129d. 31111126

[9] Pogorelova TE, Ryabchenko LE, Sunzov NI, Yanenko AS. 1996. Cobalt-dependent transcription of the nitrile hydratase gene in *Rhodococcus rhodochrous* M8. FEMS Microbiol Lett 144:191–195. doi:10.1111/j.1574-6968.1996.tb08529.x.

[10] Frederick J, Hennessy F, Horn U, de la Torre Cortés P, van den Broek M, Strych U, Willson R, Hefer CA, Daran J-MG, Sewell T, Otten LG, Brady D. 2020. The complete genome sequence of the nitrile biocatalyst *Rhodococcus rhodochrous* ATCC BAA-870. BMC Genomics 21:3. doi:10.1186/s12864-019-6405-7. 31898479 PMC6941271

[11] Martin M. 2011. Cutadapt removes adapter sequences from high-throughput sequencing reads. EMBnet J 17:10–12. doi:10.14806/ej.17.1.200.

[12] Koren S, Walenz BP, Berlin K, Miller JR, Bergman NH, Phillippy AM. 2017. Canu: scalable and accurate long-read assembly via adaptive *k*-mer weighting and repeat separation. Genome Res 27:722–736. doi:10.1101/gr.215087.116. 28298431 PMC5411767

[13] Kolmogorov M, Yuan J, Lin Y, Pevzner PA. 2019. Assembly of long, error-prone reads using repeat graphs. Nat Biotechnol 37:540–546. doi:10.1038/s41587-019-0072-8. 30936562

[14] Li H. 2013. Aligning sequence reads, clone sequences and assembly contigs with BWA-MEM. arXiv 1303.3997 [q-bio.GN]. https://arxiv.org/abs/1303.3997.

[15] Walker BJ, Abeel T, Shea T, Priest M, Abouelliel A, Sakthikumar S, Cuomo CA, Zeng Q, Wortman J, Young SK, Earl AM. 2014. Pilon: an integrated tool for comprehensive microbial variant detection and genome assembly improvement. PLoS One 9:e112963. doi:10.1371/journal.pone.0112963. 25409509 PMC4237348

[16] Hunt M, Silva ND, Otto TD, Parkhill J, Keane JA, Harris SR. 2015. Circlator: automated circularization of genome assemblies using long sequencing reads. Genome Biol 16:294. doi:10.1186/s13059-015-0849-0. 26714481 PMC4699355

[17] Tatusova T, DiCuccio M, Badretdin A, Chetvernin V, Nawrocki EP, Zaslavsky L, Lomsadze A, Pruitt KD, Borodovsky M, Ostell J. 2016. NCBI Prokaryotic Genome Annotation Pipeline. Nucleic Acids Res 44:6614–6624. doi:10.1093/nar/gkw569. 27342282 PMC5001611

